# Determinants of intermittent preventive treatment of malaria during pregnancy (IPTp) utilization in a rural town in Western Nigeria

**DOI:** 10.1186/1742-4755-9-12

**Published:** 2012-08-13

**Authors:** Olorunfemi E Amoran, Adebayo A Ariba, Christy A Iyaniwura

**Affiliations:** 1 Department of Community Medicine and Primary Care, College of Health Sciences Olabisi Onabanjo University Teaching Hospital, Sagamu, Nigeria; 2 Department of Community Medicine and Primary Care, Olabisi Onabanjo University Teaching Hospital, Sagamu, Nigeria

**Keywords:** IPTp utilization, Determinants, Rural Nigeria, Malaria in Pregnancy

## Abstract

**Background:**

Malaria infection in pregnancy is a major risk factor for maternal and child death, and substantially increases the risk of miscarriage, stillbirth and low birthweight. The aim of this study therefore is to assess the prevalence and determinants of Intermittent preventive treatment of Malaria [IPTp] utilization by pregnant women in a rural town in Western Nigeria.

**Methods:**

This study is an analytical cross-sectional study. All pregnant women that were due for delivery and were attending the three primary health care center in Sagamu town, Nigeria within a 2 months period were recruited into the study. A semi- structured questionnaire was used to collect relevant information.

**Results:**

A total of 255 pregnant women were recruited into the study. The mean age of respondents was 28.07 ± 5.12 years. The mean parity and booking age was 2.7 ± 1.67 and 4.42 ± 1.7 months respectively. The prevalence of Malaria attack in the last 3 months was 122(47.8%). Only 107/255 (40.4%) practice IPTp for malaria prevention during the current pregnancy, with only 14.6% of them taking the second dose during pregnancy as recommended. Chloroquine [27.1%] was the most frequently used medication for the treatment of Malaria in Pregnancy. Early booking age [OR = 1.11, C.I = 0.61–2.01], adverse last pregnancy outcome [OR = 1.23, C.I = 0.36–4.22], and parity [OR = 1.87, C.I = 0.25–16.09] were not statistically significantly associated with IPTp utilization. The only predictor of IPTp use was the knowledge of prophylaxis for malaria prevention [OR = 2.47, C.I = 1.06–3.52] using multivariate analysis.

**Conclusion:**

The study concludes that most women who attend ANC in rural areas in Nigeria do not receive IPTp as expected. A major determinant of utilization of IPTp among the study population was the knowledge of prophylaxis for malaria prevention. This study highlights the importance of health education of the pregnant women in increasing IPTp uptake despite the regular drug stock out at the facility level in rural areas in low resource countries.

## Background

The greatest impact of the malaria burden is felt in sub-Saharan Africa where up to 90% of the global deaths due to the disease occur [1,2] and where populations have been severely impoverished while trying to combat the disease for several decades [3,4]. About 250 million cases of malaria are recorded each year, with an estimated associated annual toll of 781,000 deaths [5]. The majority of the burden of disease caused by malaria is borne by the populations living in the highly endemic areas of sub-Saharan Africa. Within these areas, the populations at highest risk are pregnant women and infants [6].

Malaria infection in pregnancy is a major risk factor for maternal and child health, and substantially increases the risk of miscarriage, stillbirth and low birth weight [3,7]. Pregnant women are especially susceptible to the disease in areas of stable transmission. In these areas, malaria is estimated to affect 30 million pregnancies annually [5]. The transient depression of immunity to allow for development of the fetus is one of the reasons adduced for the increased susceptibility of pregnant women to malaria [8]. It is estimated that 18% of severe anaemia in pregnancy is secondary to malaria [3]. In Sub-Saharan Africa alone an estimated 200,000 to 500,000 pregnant women develop severe anaemia [3]. In a study in rural Kenya, the prevalence of anaemia in pregnancy was found to be 75.6% among primigravidae [9]. Explanations for malarial anaemia include direct destruction of a large number of infected erythrocytes as the life cycle of the Plasmodium requires that it multiplies within the infected erythrocytes and ultimately cause them to burst [9].

Nigeria has adopted the policy recommended by WHO protecting the women during pregnancy using intermittent preventive treatment (IPTp) with one curative regimen of sulphadoxine-pyrimethamine (SP) (1,500 mg sulphadoxine and 75 mg pyrimethamine), at least twice during pregnancy, once during the 2^nd^ trimester, and then at least one month apart since the year 2005. Intermittent Preventive Treatment of Malaria in Pregnancy (IPTp) involves giving a curative treatment dose of an effective anti-malaria drug at predefined intervals during pregnancy. Anti-malaria chemoprophylaxis for pregnant women living in endemic areas has been recommended for many years but the practice has been limited to the use of chloroquine and pyrimethamine [10,11]. IPTp with SP has proven efficacious in reducing the burden of pregnancy-associated malaria (PAM), and is currently part of the national malaria prevention programme in most African countries. WHO guidelines recommend a multi-pronged approach including both preventive and curative measures [12-14]. The Focused Antenatal Care approach recommends: 1) Focused antenatal care and health education: 2) Use of insecticide treated nets (ITNs): 3) Case management of women with features of clinical malaria: 4) Use of Intermittent Preventive Treatment of Malaria in Pregnancy (IPTp). National community survey 2008 Nigeria Demographic and Health Survey reported that only 6.5% of pregnant women had taken the recommended two doses of SP during pregnancy [15].

A recent study in Nigeria has compared three studies performed in Benin, showing that sulphadoxine-pyrimethamine given during pregnancy is effective both in reducing the low birth weight rate, as compared to chloroquine prophylaxis (10% with SP-IPTp given as national policy and 8.7% in a controlled IPTp trial [16], vs. 15.7% with chloroquine [17], and the malaria placental infection prevalence rate (11.2% vs. 2.9% and 16.7%, respectively), with a good overall compliance with the national IPTp policy [18]. Approximately one in four women show evidence of placental infection at the time of delivery, with a large fraction of infection remaining undetected and untreated [19]. The health consequences of malaria infection during pregnancy are large: malaria-induced low birth weight is estimated to account for up to 360,000 infant deaths every year [20]; overall, 11.4% of neonatal deaths and 5.7% of infant deaths in malaria-endemic areas of Africa are estimated to be caused by malaria in pregnancy [21,22]. But despite these observations pregnancy-related malaria remains a major cause of adverse pregnancy outcomes and effective access to malaria prevention in pregnancy remains limited in the country. The aim of this study therefore is to assess the prevalence and determinants of IPTp utilization by pregnant women in a rural town in Western Nigeria. Although there have been many previous studies on malaria morbidity and mortality in pregnant women and their babies, reports are scanty on the actual practice of IPTp in particular. There is paucity of information on whether the pregnant women actually use the drugs in the manner recommended by the WHO.

## Methodology

### Study design

This was a analytical cross-sectional study that sought to determine the pattern of IPTp utilization by pregnant women in Sagamu, Nigeria. All consenting pregnant women who attended antenatal clinic at least twice and were due for delivery were eligible to participate in the study.

### Study area

The study was carried out in Sagamu, the biggest town in Sagamu Local Government Area (LGA) located about midway between Lagos the commercial nerve center of Nigeria and Ibadan, the largest city in West Africa. It has an estimated land area of 20.05 km^2^. It is bounded on the West by Obafemi |Owode Local Government Area, on the east by Ikenne Local Government and by Ogijo town on the South. Sagamu has a population of 135,000 inhabitants projected from the 1991 census figure and consists mainly of Remo-speaking people of Ogun State. An estimated 40,000 of the women are of child bearing age. There are 15 political wards in the Local Government. Eleven of these are within Sagamu metropolis. Health facilities in the town include a Teaching Hospital, 42 private hospitals majority of which provide ante natal care services, 12 maternity homes and 30 registered birth attendants. Although the Local Government contains seven centres for primary health care services, only three within the metropolis were providing ANC activities at the time of the study. The others were not providing ANC due to organizational and logistic problems.

### Sampling technique

The minimum sample size required for the study was estimated to be 246 using the formula $N=P\;\left(1-P\right){\left(Z_{\alpha }/D\right)}^{2}$

where *n* is the sample size,

Z_α_ is the standard normal deviate, set at 1.96 (for 95% confidence level),

*d* is the desired degree of accuracy (taken as 0.05)

p, is the estimate of the proportion of pregnant women who practice IPTp = 6.5% [15].

A total of 47 women was calculated.

All women [a total of 255 women] who have had at least two antenatal visits and were due for delivery at the three designated primary health care centres over the period of study were eligible for the study were recruited into the study within a 2 months period.

### Data collection

Women who consented to partake in the survey were interviewed using a structured questionnaire, which was administered by four trained interviewers between February and March 2008. The interviewers were all female and had assisted in similar studies in the past. The data were collected on antenatal clinic days by the interviewers at the respective health care facilities. Completed questionnaires were scrutinized on the spot and at the end of daily field sessions for immediate correction of erroneous entry.

### Research instrument

The questionnaire was pretested among 20 women receiving antenatal care at similar health care facilities in Abeokuta. Appropriate adjustments were then made to the questionnaire to improve its internal validity. The instrument was a structured questionnaire which contained 39 items and was divided into 6 sections, namely socio-demographic;

Knowledge of malaria prophylaxis was determined by assessing the correct answer given to a set of 4 simple questions about the cause, transmission, consequences’ and the need for prophylaxis to prevent Malaria in pregnancy such as

What is the cause of Malaria?

Can Malaria be transmitted to unborn child during pregnancy?

What are the consequencies of Malaria in pregnancy [with options]?

Can sulphadoxine-pyrimethamine [fansidar] be used to prevent Malaria in pregnancy?

100% was taken as having a good knowledge of Malaria prophylaxis in pregnancy.

IPTp use was defined as those that used SP for at least once in pregnancy for prevention of Malaria.

Information and counseling on malaria during pregnancy, use of malaria prevention treatment, experience of malaria disease during pregnancy and use of insecticide treated nets were also assessed.

The socio-demographic section included information on socio-demographic data such as age, marital status, parity, number of living children, occupation of the woman and her husband, educational level completed. Others were duration of pregnancy at the time of interview, duration of pregnancy at the first antenatal visit. The other sections explored women’s knowledge and access to information on malaria.

### Criteria for inclusion

#### Subjects recruited into the study fulfilled the following criteria

· Have had at least one antenatal clinic visit before the interview. This was used as proof that they have been registered in that facility.

· Only pregnant women that were due for delivery were interviewed.

#### Criteria for exclusion

· Registering for the first time in the health facility.

· Disabilities that disallowed responses to questionnaire.

· Refusal to partake in study.

### Ethical consideration

Ethical clearance was obtained from the Olabisi Onabanjo Teaching Hospital Ethics Board. Confidentiality on candidate's information was maintained. Permission of the State Ministry of Health was obtained before the commencement of the study.

At each of the selected study site, the matron and medical officer in-charge were informed for consent and antenatal clinic day(s) before the commencement of the study. The purpose, general content and nature of the study were explained to each respondent to obtain verbal and written consent before inclusion into the study.

### Data analysis

The data were coded and entered into a computer database using EPI INFO 2002 statistical software (CDC and WHO 2002). Percentages or means and standard deviations were computed for baseline characteristics of women interviewed. Cross tabulations that identify important relationships between variables were done. The relationship between socio-demographic characteristics of the women and their use or non-use of intermittent preventive treatment of malaria during pregnancy was examined through bivariate analysis, by computing odds ratio at 95% confidence level. A *p*-value < 0.05 was considered as statistical significance.

## Result

### The socio-dermographic characteristics and obstetric history of respondents

The socio-demographic characteristics of the respondents interviewed at the various health facilities are presented below. The mean age was 28.07 ± 5.12 years. 232 (91.0%) of the respondents were married and only a few (6.7%) were single. Among the participants, 20(7.8%) of the respondents had no formal education, 45(17.6%) had primary education, 120 (47.1%) had secondary school education, 70 (31.1%) had post secondary school education. 87(34.1%) of the respondents were artisans, 15(5.9%) were students, 29(11.4%) were petty traders, 93 (38.5%) had a white collar job, while 31 (12.2%) were unemployed.

The majority of the women were multiparous while about a quarter of them were expecting their first babies. The mean parity was 2.7 ± 1.67 and the median parity was 3.0. Less than one-tenth of the respondents were grand-multips those who have never had a child and those who have no children alive were 106 (41.6%). Less than one-quarter of them had at least one child alive. Majority of the mothers (77.6%) booked between the gestational ages of 3 and 6 months with 4 months being the most frequently preferred month for initiating ante natal care. The mean booking age of the women was 4.42 ± 1.7 months. 195 women (76.5%) had experienced previous deliveries. The interval between the last child birth and current pregnancy for over a quarter of the women (26.2%) was 2 years.

### Malaria knowledge and prevention ractices

Most of the mothers, (45.9%) believes that the risk of malaria increases with pregnancy. More than half (52.9%) knew that malaria is associated with low birth weight while (45.9%) knew that malaria can cause both pregnancy loss and maternal anaemia. One hundred and fifty four (60.4%) of the respondents reported they received some information on malaria prevention during pregnancy. As depicted in Table 1 nurses and doctors were the major source of information to well over half (68.9%) of the respondents while chemists were the source of information to 7.1% of the women on malaria during pregnancy. The rest of the women who got information on malaria during pregnancy got it through other sources (such as the media, friends, faith based organization, etc.). 122(47.8%) reported that they had Malaria attack in the last 3 months. .Chloroquine [27.1%] was the most frequently used medication for the treatment of Malaria, followed by Sulphadoxine-pyrimethamine (SP) [17.1%], arthemisinin based combination therapy [13.9%] and native medication [16.4%]. Of the 255 mothers who participated in the study, only 33(12.9%) of the respondents slept under ITN during their current pregnancies.

**Table 1:** Malaria prevention practices among respondents

	**No. of respondents**	**Percent (%)**
**Type of dugs taken for the treatment of Malaria in Pregnancy**		
Chloroquine	33	27.1
Sulphadoxine-pyrimethamine (SP)	21	17.1
Arthemisinin	17	13.9
Native medication	20	16.4
Camoquine	31	25.4
**Total**	**122**	**100.0**
**Source of information about Malaria Prevention in Pregnancy**		
Nurse	54	35.1
Chemist	11	7.1
Doctor	52	33.8
Others	37	24.0
**Timing of use of IPTp**		
1 Trimester	27	26.2
2 Trimester	72	66.0
3 Trimester	8	7.8
**Total**	**107**	**100.0**
Time after initial dose second dose taken		
1^st^ week	48	46.6
2^nd^ week	15	14.6
3^rd^ week	8	7.8
4^th^ week	15	14.6
Not taken	21	16.5

### Intermittent preventive treatment of malaria

Among all the respondents 107/255 (40.4%) reported taking medications [SP] for malaria prevention [IPTp] during the current pregnancy. Majority of the mothers (84.4%) who reported to have taken drugs for malaria prevention during pregnancy took the drug at home. The remaining 13(12.6%) took the medications at the health facilities. Most (66.0%) of the women took the drugs during the second trimester of pregnancy, while only a quarter of them took the first dose of malaria preventive therapy during the first trimester. These facts are further shown in Table 1. Of the 107 women who started anti-malarial medication for prevention during pregnancy 86 (83.3%) took a second dose at various times after the initial dose with only 14.6% of them using it as recommended at 4th week following the initial dose. Reasons given for not taking SP included not been informed by health workers, afraid of safety of drug, drug stock out, did not remember to buy etc.

The utilization of IPTp was not statistically significantly associated with older age {>24 year} [OR = 1.14, C.I = 0.22–5.95], Higher level of education (above secondary school) [OR = 0.72, C.I = 0.23–2.24] and white collar job [OR = 1.05, C.I = 0.42–2.61]. However, the knowledge of prophylaxis for malaria prevention had a statistically significant relationship with use of IPTp, [OR = 3.19, C.I = 1.26–8.42]. Furthermore IPTp use was not statistically significantly associated with obstetric history such as early booking age [OR = 1.11, C.I = 0.61–2.01], adverse last pregnancy outcome [OR = 1.23, C.I = 0.36–4.22], and parity [OR = 1.87, C.I = 0.25–16.09]. This is illustrated in Tables 2 and 3.

**Table 2:** Socio-dermographic characteristics and IPTp use

	**% IPTP non users No [%]**	**% IPTP use No [%]**	**Total No [%]**	**ODDS RATIO [C.I]**
**Age**				
15–19 yrs	5 [3.4]	5 [4.7]	10 [3.9]	1.00
20–24 yrs	28 [18.9]	21 [19.6]	49 [19.2]	1.33 [0.28–6.29]
25–34 yrs	99 [66.9]	67 [62.6]	166 [65.1]	1.48 [0.35–6.18]
>35 yrs	16 [10.8]	14 [13.1]	30 [11.8]	1.14 [0.22–5.95]
Total	148 [100.0]	107 [100.0]	255 [100.0]	
**Educational level**				
Nil	13 [8.8]	7 [6.5]	20 [8.9]	1.00
Primary	21 [14.2]	24 [22.4]	45 [20.0]	0.47 [0.14–1.58]
Secondary	74 [50.0]	46 [43.0]	120 [53.3]	0.87 [0.29–2.55]
Tertiary	40 [27.0]	30 [28.0]	70 [31.1]	0.72 [0.23–2.24]
**Occupation**				
Unemployed	19 [12.8]	12 [11.2]	31 [12.2]	1.00
Students	**8 [5.4]**	7 [6.5]	15 [5.9]	0.72 [0.17–2.98]
Traders	18 [12.2]	11 [10.3]	29 [11.4]	1.03 [0.32–3.33]
Artisans	45 [30.4]	42 [39.3]	87 [34.1]	0.68 [0.27–1.69]
White collar jobs	58 [39.2]	35 [32.7]	93 [36.5]	1.05 [0.42–2.61]
**Knowledge of Malaria prophylaxis in Pregnancy**				
Adequate	121 [81.8]	100 [93.5]	221 [86.7]	3.19 [1.26–8.42]
Not-adequate	27 [18.2]	7 [6.5]	34 [13.3]	1.00

**Table 3:** Obstetric history and IPTp use

	**% IPTP non users No [%]**	**% IPTP use No [%]**	**Total No [%]**	**ODDS RATIO [C.I]**
**Booking age**				
Ist trimester	42 [28.4]	33 [30.8]	75 [29.4]	1.00
2^nd^ trimester	92 [62.2]	65 [60.8]	157 [61.6]	1.11 [0.61–2.01]
3^rd^ trimester	14 [9.4]	9 [8.4]	23 [9.0]	1.22 [0.43–3.53]
**Last Pregnancy Outcome**				
Nil Birth	19 [12.8]	21 [19.6]	40 [15.7]	1.00
Still Birth	10 [6.8]	9 [8.4]	19 [7.5]	1.23 [0.36–4.22]
Preterm	0 [0.0]	2 [1.9]	2 [0.8]	0.00 [0.00–5.12]
Live Birth	119 [80.4]	75 [70.1]	194 [76.1]	1.75 [0.84–3.67]
**Parity**				
0	31 [20.9]	29 [2.7]	60 [23.5]	1.00
1–2	73 [49.3]	56 [52.3]	129 [50.6]	1.22 [0.63–2.36]
3–4	40 [27.0]	20 [18.7]	60 [23.5]	1.87 [0.84–4.19]
>5	4 [2.7]	2 [1.9]	6 [2.4]	1.87 [0.26–16.09]

### Predictors of utilization of IPTp

Table 4 shows the adjusted odds ratio for factors associated with utilization of IPTp among the study population. The only predictor of IPTp use was the knowledge of prophylaxis for malaria prevention [OR = 2.47, C.I = 1.06–3.52] after controlling for the effect of confounders using multivariate analysis.

**Table 4:** Predictors of utilization of IPTp

**Characteristics**	**Adjusted Odds ratio [C.I]**
**Booking age**	
Ist trimester	1.00
2^nd^ trimester	1.51 [0.82–2.75]
3^rd^ trimester	1.41 [0.74–5.88]
**Last Pregnancy Outcome**	
Nil Birth	1.00
Still Birth	1.09 [0.72–3.54]
Preterm	0.00 [undefined]
Live Birth	1.53 [0.76–3.22]
**Parity**	
0	1.00
1–2	1.43 [0.51–4.27]
3–4	1.76 [0.65–5.68]
>5	1.92 [0.48–9.37]
**Knowledge of Malaria prophylaxis in Pregnancy**	
Adequate	2.47 [1.06–3.52]
Not-adequate	1.00

## Discussion

Utilization [40.4%] of SP (the drug recommended by the policy for IPTp) for preventive treatment of malaria with 14.6% taking the second dose at the appropriate time was very low among the study population and only 12.9% sleeping under ITN. This observation has been made by several other studies in Nigeria and other part of Africa [15,23,24]. Even lower prevalence was reported in the National community survey 2008 Nigeria Demographic and Health Survey with only 11.8% of pregnant women slept under an ITN, and only 6.5% of pregnant women had taken the recommended two doses of SP during pregnancy [15]. This observation has been attributed to fear of the safety of SP during pregnancy on the part of the health workers and also unavailability of the drugs at the facility due to stock out. This indicates that programs that will be geared towards increasing the knowledge and awareness of the importance of IPTp and other preventive measures for Malaria prevention should be introduced among this vulnerable population in the rural areas in Africa. This will definitely lead to reduction in maternal mortality since most of the death comes from this rural environment.

The reported prevalence of malaria among the study population was about 48%. This is similar to report by several other studies [23,24]. This is not surprising since most of the women did not benefit from proper IPTp, accordingly, the prevalence of malaria in pregnancy remains high. Of note is the finding that chloroquine was still the most frequently used drug for treating malaria during pregnancy by these women. More worrisome is the high proportion still taking native medication. This calls for the proper implementation of the National policy on Malaria treatment and prevention which has been shown to significantly reduce malaria related morbidity and deaths in pregnant women [12,13].

The average gestational age of booking for ANC by participants in this study was 4 months. While a few of them booked early the number of mothers booking increased gradually, and got to a peak during the second trimester and then gradually declined as pregnancy neared term. This pattern of ANC booking is a deviation from the regular one in this environment which is characterized by late booking by majority of mothers [24,25], and clearly reflects an interaction of several factors, chief among which is the high level of education of the women. Their maturity in terms of age, and the fact that many of them have had contact with ANC services before (most were multips) were also important contributors to this pattern. It is of concern therefore that despite their presenting at a good time for information on, and application of IPTp most of these mothers reported that they neither received information on this policy nor took the treatment. This calls for a review of the implementation of this policy which has been shown to significantly reduce malaria related morbidity and deaths in pregnant women. In a related work in Nigeria and Tanzania [26,27] it was found that more than half of pregnant mothers first attendees at ANC do not receive IPTp on their first visit. This study suggests that the PHC health workers should be trained on the importance of IPTp in the prevention of Malaria and its complication and the need to strictly implement this policy at the PHC.

A major determinant of utilization of IPTp among the study population was the knowledge of prophylaxis for malaria prevention. For pregnant women to use IPTp properly they must be well informed about the dangers of pregnancy-related malaria and receive the appropriate therapy at the right time during pregnancy. The focus on community directed interventions in the design of Preventive intervention is essential. This highlights the importance of health education at the community level in the delivery of health services in general, and preventive health measures in particular. Several studies have reported similar experiences [28-35]. While the involvement of community workers does not necessarily mediate socioeconomic differences within communities [36], the overall health improvements achievable through community based interventions appear large [29,37]. Health education of the community will increase IPTp uptake despite the regular drug stock out at the facility level.

Our study has certain limitations. The study findings are limited in terms of overall generalization and impact since the study might also have been faced with recall bias. Furthermore the study was conducted at the health facilities, and this may lead to selection bias as the situation of pregnant women who do not attend the health facility is not known. Despite these limitations, we believe that our data provide useful information for the assessment and implementation of IPTp in Nigeria and will also inform policy decision in Nigeria and other low income countries.

## Conclusion

The study concludes that most women who attend ANC in rural areas in Nigeria do not receive IPTp as expected. A major determinant of utilization of IPTp among the study population was the knowledge of prophylaxis for malaria prevention. Uptake of IPTp can be significantly improved in rural areas if backed up with appropriate health education intervention. This study highlights the importance of community health education in the delivery of health services in general, and preventive health measures in particular.

## Competing interest

The authors declare that they have no competing interests.

## Authors’ contribution

AAA conceived the study and participated in its design, CAI participated in its design and data collection, AOE participated in the analysis and helped to draft the manuscript. All authors read and approved the final manuscript.
